# Broad-Spectrum Virucidal Activity of Nitric Oxide Nasal Spray (NONS) Against SARS-CoV-2 Variants and Major Respiratory Viruses

**DOI:** 10.3390/v18010091

**Published:** 2026-01-09

**Authors:** James Martins, Selvarani Vimalanathan, Jeremy Road, Chris Miller

**Affiliations:** 1 SaNOtize Research & Development Corp., Vancouver, BC V6P 6T3, Canada; jmartins@sanotize.com (J.M.); chris@sanotize.com (C.M.); 2 Department of Pathology and Laboratory Medicine, University of British Columbia, Vancouver, BC V6T 2B5, Canada; 3 Respiratory Medicine, University of British Columbia, Vancouver, BC V6T 1Z4, Canada

**Keywords:** nitric oxide, nitric oxide nasal spray, respiratory viruses, *Coronaviridae*, *Orthomyxoviridae*, *Paramyxoviridae*, *Picornaviridae*, virucidal activity, intranasal therapy, respiratory virus control

## Abstract

Respiratory viruses such as SARS-CoV-2, influenzas A and B, respiratory syncytial virus (RSV), human metapneumovirus (hMPV), human parainfluenza virus type 3 (HPIV-3), and rhinoviruses remain major causes of global morbidity. Their rapid evolution, high transmissibility, and limited therapeutic options, together with the absence of approved vaccines for several pathogens, highlight the need for broad-acting and pathogen-independent antiviral strategies. Nitric oxide exhibits antiviral activity through redox-dependent mechanisms, including S-nitrosylation of cysteine-containing viral proteins and disruption of redox-sensitive structural domains. Clinical studies conducted during the SARS-CoV-2 pandemic demonstrated that a nitric oxide nasal spray (NONS) rapidly reduced nasal viral load and transmission. In this study, we evaluated the in vitro virucidal activity of the NONS against a panel of clinically relevant respiratory viruses representing four major virus families. Virus suspensions of approximately 10^4^ CCID_50_ were exposed to a full-strength NONS for contact times ranging from 5 s to 2 min at room temperature, followed by neutralization and quantification of residual infectivity using endpoint dilution assays. The NONS rapidly reduced viral infectivity across all viruses tested, achieving >3 log_10_ reductions within 2 min. SARS-CoV-2 variants including Alpha, Beta, Gamma, Delta, Omicron BA.1, and XBB 2.0 were reduced to levels at or below the assay detection limit within 30 s to 2 min. Influenza A and B viruses showed the fastest loss of infectivity, reaching detection limits within 10–15 s. RSV, hMPV, HPIV-3, and human rhinovirus 14 were similarly inactivated within 1–2 min. These findings demonstrate that the NONS exhibits rapid and broad-spectrum virucidal activity against diverse respiratory viruses and supports its potential role in pandemic preparedness but also seasonal use.

## 1. Introduction

Endogenous nitric oxide (NO) is a gaseous signaling molecule produced by nitric oxide synthases, with essential roles in immune regulation, endothelial function, and antimicrobial defense. Beyond its physiological functions, exogenously delivered NO has demonstrated broad-spectrum antiviral activity through mechanisms such as S-nitrosylation of viral proteins, disruption of envelope integrity, and interference with host–virus interactions. These properties support its therapeutic potential in targeting the upper respiratory tract, particularly the nasal cavity, where respiratory viruses initially colonize and replicate. Given that the nasal epithelium is a major site of viral replication, shedding and transmission, early intervention with intranasal NO therapies such as NONSs may reduce infection and transmission and help prevent viral spread to the lower respiratory tract [1,2,3].

Respiratory viruses, such as SARS-CoV-2, influenza A viruses, influenza B virus, respiratory syncytial virus (RSV), human metapneumovirus (hMPV), human parainfluenza virus type 3 (HPIV-3), and rhinoviruses, remain major contributors to global respiratory morbidity and mortality each year. These RNA viruses are capable of causing a wide range of symptomatic clinical outcomes, from mild upper respiratory tract symptoms to severe pneumonia and acute respiratory distress syndrome (ARDS) [4,5,6]. Their high mutation rates promote rapid antigenic drift, undermining vaccine effectiveness and facilitating immune escape [7]. SARS-CoV-2 variants such as Omicron contain multiple spike protein mutations that alter viral structure and reduce susceptibility to neutralizing antibodies [8]. Reinfections therefore remain common due to waning immunity and ongoing emergence of immune-evasive variants [9,10]. Moreover, several of these pathogens still lack approved vaccines emphasizing the need for effective broad-spectrum antiviral strategies.

The resurgence of highly pathogenic avian influenza (HPAI) H5N1 further heightened concern about viral contagion and spillover risk. The currently circulating clade 2.3.4.4b lineage has caused extensive mortality in wild and domestic birds and has increasingly spilled over into mammals such as minks, sea lions, cats, and dairy cattle, suggesting raising concern regarding zoonotic potential [11,12,13]. Although human infections remain relatively rare, the high case-fatality rate and the emergence of adaptive mutations in hemagglutinin and polymerase genes raise concern about mammalian adaptation and sustained transmission. Because these viruses can cross species barriers and evolve rapidly, they highlight the urgency of developing antivirals that act through pathogen-independent mechanisms.

Evidence of zoonotic emergence of viruses such as SARS-CoV, MERS-CoV, H5N1, and the 2009 H1N1 influenza pandemic illustrates the continuing risk of cross-species transmission [14,15,16]. Collectively, these events demonstrate the ability of respiratory viruses to trigger global outbreaks and emphasize the importance of proactive surveillance and innovative antiviral development that remain effective across diverse viral families.

Because elevated nasopharyngeal viral loads are associated with increased transmissibility [17,18], NONS was developed as a self-administered intranasal spray that delivers nitric oxide within a protective gel barrier directly onto the nasal mucosa. This allows antiviral activity at the interface where both newly introduced and endogenously replicated virions are present.

In vitro studies have shown >99% reductions in viral load within two min of exposure, and human clinical trials during the SARS-CoV-2 pandemic demonstrated rapid decreases in viral load, transmission and symptoms with no serious adverse events [19,20,21].

Two randomized, placebo-controlled trials showed that early treatment with NONS significantly accelerated viral clearance, shortening infection duration by approximately 50% compared with saline placebo and improving symptom resolution [19,20]. Real-world exposure studies are consistent with substantially lower infection rates among individuals using NONS compared with untreated controls. In a university exposure study, students who used NONS for 10 days following confirmed close-contact exposure (*n* = 203) had substantially lower infection rates than untreated exposed controls (*n* = 422), with only 6% testing positive compared with 24% in the control group [21].

A Phase 3 randomized, double-blind, placebo-controlled trial also demonstrated that NONS significantly accelerated viral clearance in high-risk outpatients, with RT-PCR test turning negative a median of four days earlier than in the placebo group (three vs. seven days), and 82.8% of treated individuals testing negative by Day 8 compared with 66.7% of placebo recipients [19]. Earlier studies likewise showed rapid symptom improvement and reductions in viral load within 24–48 h of treatment, with consistent activity across multiple circulating SARS-CoV-2 variants [20].

NONS is currently approved in India as FabiSpray and is marketed internationally as VirX^®^ or Enovid^™^, and as NOWonder^TM^ as a nasal cleanser. Its pathogen-independent mechanism, rapid virucidal activity within a gel barrier, and favorable safety profile make it a practical option to reduce viral transmission, particularly among vulnerable populations.

In this study, we evaluated NONS beyond SARS-CoV-2 to include additional clinically relevant respiratory viruses such as influenza A (H1N1, H3N2 and H5N1), influenza virus B, rhinovirus, RSV, hMPV and HPIV-3 to assess its broader potential as a versatile antiviral platform. This comprehensive assessment is important because most of these viruses replicate initially in the upper respiratory tract before disseminating to the lungs. Together, they contribute substantially to community transmission and morbidity and therefore remain as important targets for broad-spectrum antiviral development.

## 2. Materials and Methods

### 2.1. Virus, Media, and Cells

The virucidal efficacy of the nitric oxide nasal formulation (NONS) was evaluated against a panel of respiratory viruses representing both enveloped and non-enveloped RNA viruses. The following virus strains were used: SARS-CoV-2 Alpha (B.1.1.7), Beta (B.1.351), Gamma (P.1), Delta (B.1.617.2), Epsilon (B.1.427), Omicron (B.1.1.529), and Omicron XBB.2.0 variants, and human metapneumovirus (hMPV), all propagated in Vero E6 cells (ATCC); influenza A viruses H1N1 (A/California/07/2009), H3N2 (A/Perth/16/2008), and H5N1 (A/Duck/MN/1525/1981), and influenza B virus in MDCK cells (Sigma-Aldrich MilliporeSigma, Burlington, MA, USA); respiratory syncytial virus (RSV A2 strain) and human parainfluenza virus type 3 (HPIV-3) in MA-104 Clone 1 cells (ATCC, Manassas, VA, USA); and human rhinovirus 14 (HRV-14) in HeLa-Ohio cells (Sigma-Aldrich, Burlington, MA, USA). A549 cells (ATCC, Manassas, VA, USA) were used for cytotoxicity testing.

All cell lines were maintained under standard conditions (37 ± 2 °C, 5% CO_2_, humidified atmosphere) for virus propagation and endpoint titration. Viral titers for untreated controls ranged from 3.3 to 4.7 log_10_ CCID_50_ per 0.1 mL, corresponding to approximately 2 × 10^4^ to 5 × 10^5^ PFU/mL.

#### Nitric Oxide Nasal Spray (NONS) Formulation

NONS consists of three components that are mixed immediately before use to generate a nitric-oxide–releasing gel: (i) a nitrite-donor solution, (ii) an acidified gel matrix containing buffering and stabilizing excipients, and (iii) a small amount of preservative within each chamber to maintain long-term shelf life. Mixing the components results in controlled, localized release of nitric oxide at the nasal mucosal surface. The excipients function only as stabilizers and gelling agents and do not possess antiviral activity at the concentrations used. Control experiments confirmed that excipient mixtures lacking nitric oxide did not measurably reduce viral titers under the assay conditions. Proprietary identifiers and exact ratios are not disclosed due to regulatory agreements; however, all functional constituents are described.

### 2.2. General Experimental Conditions

All experiments were performed in triplicate unless otherwise stated. Unless specified, all cell incubations were carried out at 37 °C in a humidified 5% CO_2_ incubator.

### 2.3. Cell Culture and Cytotoxicity Assessment

A549 cells were seeded into 96-well plates at a density of 1 × 10^4^ cells per well in Dulbecco’s Modified Eagle Medium (DMEM) supplemented with 5% fetal bovine serum (FBS) and incubated for 24 h at 37 °C in a humidified 5% CO_2_ atmosphere. The culture medium was then replaced with 100 µL of NONS at final concentrations of 12.5%, 25%, 50%, or 100%, and cells were exposed for 30 min. Following treatment, cells were washed and incubated in fresh DMEM for an additional 24 h. Cell viability was assessed using the XTT cell proliferation assay kit (ATCC). Activated XTT reagent (50 µL) was added to each well and incubated for 2 h at 37 °C. Absorbance was measured at 475 nm using a microplate reader, with background correction performed using cell-free wells containing medium and XTT reagent. Cell viability was expressed as a percentage relative to untreated control cells.

### 2.4. Virucidal Assay and Infectivity Evaluation

Samples A and B were provided as separate liquid components mixed in equal volumes immediately before testing to generate the 100% NONS formulation. NONS was evaluated against SARS-CoV-2 (wild-type and variants), influenza A (H1N1, H3N2, and H5N1), influenza B, RSV, HRV-14, hMPV, and HPIV-3.

Each virus stock was titrated and 10^4^ CCID_50_ aliquots were added to triplicate tubes containing the 100% NONS formulation at a ratio of 1 part virus to 9 parts NONS (10% virus: 90% formulation). Mixtures were incubated at room temperature (22 ± 2 °C) with samples taken at 5, 10, 15, 30 s, 1 min, and 2 min. Each sample was immediately neutralized by a 1:10 dilution in DMEM containing 2% FBS and titrated on virus-permissive cell lines using standard endpoint-dilution assays.

Neutralization controls confirmed that the neutralized formulation exhibited no residual antiviral activity. Neutralization controls also verified that virus inactivation did not continue beyond the specified contact time and that residual formulation in the titration step did not suppress viral growth. This assay follows standardized virucidal testing protocols in which the primary outcome is log-reduction rather than absolute residual titer; therefore, reductions apply proportionally across a range of starting viral loads.

Toxicity controls (formulation + cells, without virus) and neutralization controls (formulation neutralized before virus addition) were included for all experiments. Toxicity controls demonstrated ≥90% cell viability following exposure to neutralized formulation, and neutralization controls confirmed that, once NONS was neutralized, no further antiviral effect occurred during titration.

Virus control samples (virus + water) served as negative controls. Seventy percent ethanol served as the positive virucidal control and was selected because it is a widely accepted reference agent in standardized virucidal testing guidelines for validating assay performance.

To assess infectivity after treatment, NONS-treated, ethanol-treated, Phosphate-Buffered Saline (PBS)-treated, water-treated, media-treated viral preparations were diluted 1:10 in DMEM with 2% FBS and used to infect the appropriate virus-permissive cell line for each virus (e.g., Vero E6 for SARS-CoV-2 and hMPV; MDCK for influenza A and B; MA 104 for RSV and HPIV-3; HeLa-Ohio cells for HRV-14). After 48 h, or after the optimal incubation period for each virus, viral infectivity was assessed using cell-viability assays and cytopathic-effect (CPE) evaluation.

### 2.5. Cell Viability Following Infection

Cell viability after infection was quantified using the CellTiter-Glo Luminescent Cell Viability Assay (Promega, Madison, WI, USA; Cat. No. G7571). After removing supernatants, 50 μL fresh media and 50 μL CellTiter-Glo reagent were added to each well. Plates were mixed gently and incubated for 20 min in the dark at room temperature. Luminescence was measured using a SpectraMax M5 microplate reader (Molecular Devices, San Jose, CA, USA) with a 1000 ms integration time.

### 2.6. Virus Quantification

Residual infectious virus in neutralized samples was quantified using standard endpoint-dilution assays. Neutralized samples were serially diluted tenfold and inoculated onto monolayers of the appropriate virus-permissive cell line. Plates were monitored for cytopathic effect (CPE), and infectious titers were calculated using the Reed–Muench method to determine CCID_50_. Log-reduction values (LRVs) were calculated relative to the virus-only (water-treated) control.

### 2.7. Statistical Analysis

All experiments were performed in triplicate unless otherwise stated. Viral titers are expressed as log_10_ CCID_50_ per 0.1 mL. Log-reduction values were calculated relative to virus-only controls, and virucidal outcomes were interpreted according to predefined log-reduction thresholds, which represent the primary endpoint of the assay.

**Figure 1:**
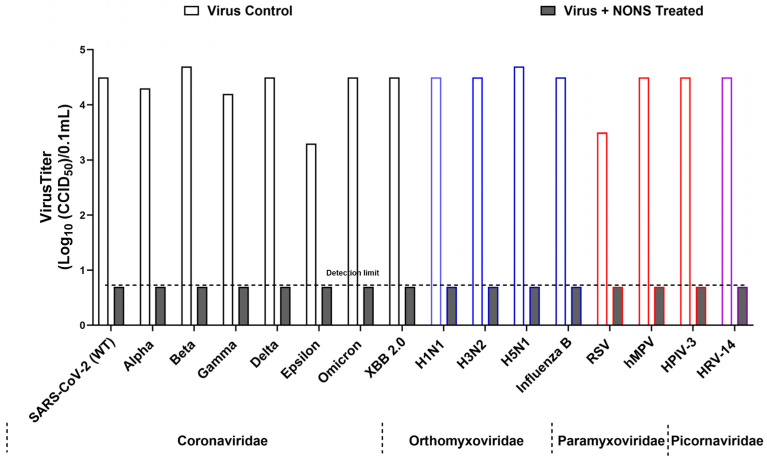
Broad-spectrum virucidal activity of nitric oxide nasal spray (NONS) against major respiratory viruses. Virus titers (log_10_ CCID_50_/0.1 mL) were measured for untreated controls and NONS-treated samples after 2 min of exposure at room temperature. Values are shown as mean titers from independent experiments. For several viruses, all replicate titers in the NONS-treated group were identical and at the assay detection limit, resulting in SD = 0; therefore, inferential statistical testing was not applied. The dashed horizontal line indicates the detection limit (0.7 log_10_ CCID_50_/0.1 mL). Viruses are grouped by family (*Coronaviridae*, *Orthomyxoviridae*, *Paramyxoviridae*, *Picornaviridae*), and colored bar outlines are used to denote families for visual clarity. NONS treatment produced pronounced reductions in infectious virus compared with untreated controls.

For Figure 1, results are reported as mean values from three independent experiments. For several viruses, all replicate titers in the NONS-treated group were identical and at the assay detection limit, resulting in SD = 0; therefore, inferential statistical testing (e.g., Student’s *t*-tests) was not applied to these data. Where applicable in other experiments, comparisons between treated and control samples were evaluated using two-tailed Student’s *t*-tests, and data are presented as mean ± SD. Data analysis and graph preparation were performed using GraphPad Prism version 10.6.1.

## 3. Results

### 3.1. Cytotoxicity Assessment of NONS

To evaluate the cytotoxicity of the NONS, A549 cells were treated with increasing concentrations ranging from 12.5% to 100% for 30 min. Cell viability was assessed using the XTT assay. A dose-dependent decrease in viability was observed, with approximately 92% of cells remaining viable at the 100% NONS concentration. This concentration was therefore selected for all subsequent antiviral assays.

### 3.2. Virucidal Activity of NONS Against SARS-CoV-2 Variants (Coronaviridae)

The virucidal efficacy of the NONS was evaluated against multiple SARS-CoV-2 variants, including wild-type, Alpha (B.1.1.7), Beta (B.1.351), Gamma (P.1), Delta (B.1.617.2), Epsilon (B.1.429), Omicron (BA.1), and XBB 2.0. Virus suspensions (~10^4^ CCID_50_/0.1 mL) were incubated with the NONS, with samples subjected to contact times of 30 s to 2 min at room temperature (22 ± 2 °C).

**Figure 2:**
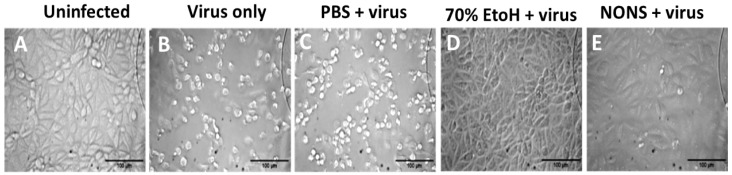
Prevention of SARS-CoV-2-induced cytopathic effects in Vero E6 cells by nitric oxide nasal spray (NONS). Bright-field micrographs show the morphological appearance of Vero E6 cells following 48 h incubation under different treatment conditions: (**A**) uninfected control cells showing intact monolayer morphology; (**B**) Virus-infected cells showing extensive cytopathic effects, including cell rounding and detachment. (**C**) Virus + PBS control showing cytopathic effects similar to the virus-only condition; (**D**) cells exposed to 70% ethanol prior to infection showing preserved monolayer, confirming complete viral inactivation; (**E**) cells exposed to NONS-treated virus exhibiting morphology comparable to uninfected controls, indicating prevention of virus-induced cytopathic effects. Scale bar = 100 µm.

All variants showed complete inactivation, with titers reduced from 3.3–4.7 log_10_ CCID_50_ to below the detection limit of 0.7 log_10_ CCID_50_, corresponding to log reduction values (LRVs) greater than 3.0–4.0 (>99.9% reduction in infectivity). The wild-type and Delta variants were inactivated within 30 s, while Beta, Gamma, Epsilon, and Omicron required up to 2 min. Ethanol (70%) yielded comparable inactivation levels. Control assays confirmed that the observed effects were attributable to direct virucidal activity. These findings demonstrate that NONS exhibits rapid and consistent efficacy across SARS-CoV-2 variants of concern (Figure 1 and Figure 2, Table 1). Toxicity and neutralization controls verified that decreased infectivity resulted from direct virucidal action rather than cytotoxicity or residual formulation effects.

### 3.3. Virucidal Activity of NONS Against Influenza Viruses (Orthomyxoviridae)

The virucidal activity of NONS was further assessed against influenza A subtypes H1N1, H3N2, and H5N1, as well as influenza B. Viral suspensions (~10^4^ CCID_50_/0.1 mL) were treated with NONS for up to 2 min at room temperature, and titers were quantified on MDCK cells using endpoint dilution.

For all influenza strains, NONS treatment reduced titers from 4.1–4.7 log_10_ CCID_50_ to below the detection limit of 0.7 log_10_ CCID_50_, corresponding to LRVs > 3.4–4.0 and >99.9% reduction in infectivity. Complete inactivation was observed within 15 s for influenza A (H1N1, H3N2, H5N1) and B, highlighting these viruses as highly sensitive to nitric-oxide-mediated inactivation.

Ethanol produced similar reductions but required longer exposure, confirming that NONS exerts potent and rapid virucidal activity suitable for intranasal application (Table 1 and Figure 1).

### 3.4. Virucidal Activity of NONS Against RSV, hMPV, and HPIV-3 (Paramyxoviridae)

To evaluate whether NONS retains activity against other major respiratory pathogens, viruses from the *Paramyxoviridae* family including respiratory syncytial virus (RSV A2), human metapneumovirus (hMPV), and human parainfluenza virus type 3 (HPIV-3) were tested.

All three viruses were completely inactivated following 2 min of exposure to 100% NONS. RSV titers declined from 3.5 to <0.7 log_10_ CCID_50_ (LRV > 2.8; >99.8% reduction), while hMPV and HPIV-3 exhibited LRVs > 3.1–3.4 (>99.9% reduction). Toxicity and neutralization controls confirmed that the loss of infectivity resulted from direct virucidal action. These findings extend the antiviral efficacy of nitric oxide to key pediatric respiratory pathogens.

### 3.5. Virucidal Activity of NONS Against Human Rhinovirus (Picornaviridae)

Human rhinovirus 14 (HRV-14), a non-enveloped RNA virus known for resistance to many disinfectants, was used to assess whether NONS is effective against non-enveloped viruses. The control titer (4.5 log_10_ CCID_50_) was reduced to below 0.7 log_10_ CCID_50_ after 2 min of NONS treatment (LRV > 3.8; >99.98% reduction).

In contrast, ethanol achieved only partial inactivation, reducing titers to ~4.0 log_10_ CCID_50_ (LRV 0.5). Under these assay conditions, NONS demonstrated broader virucidal activity, including activity against the non-enveloped virus HRV-14 (Table 1; Figure 1).

## 4. Discussion

This study demonstrates the broad-spectrum virucidal activity of a nitric oxide nasal spray (NONS) against a comprehensive panel of clinically relevant respiratory viruses, including SARS-CoV-2 variants, influenza A and B (including highly pathogenic avian influenza H5N1) viruses, respiratory syncytial virus (RSV), human metapneumovirus (hMPV), parainfluenza virus type 3 (HPIV-3), and human rhinovirus 14 (HRV-14). These viruses collectively represent four major respiratory virus families: *Coronaviridae*, *Orthomyxoviridae*, *Paramyxoviridae*, and *Picornaviridae*, each with distinct structural genomic and characteristics. The rapid and consistent viral inactivation observed across both enveloped and non-enveloped viruses supports a pathogen-independent mechanism of NONS, most likely involving S-nitrosylation of cysteine residues within viral proteins, resulting in conformational instability and impaired host cell attachment, entry or replication [2,22,23].

It is important to note that NONS delivers exogenous nitric oxide through a controlled chemical reaction within a sprayed gel to the surface of the nasal mucosa rather than relying on endogenous nitric oxide synthase (NOS) pathways. Endogenous nitric oxide is generated by nitric oxide synthase enzymes and participates in vascular regulation, neurotransmission, and innate immune defense. However, excessive nitric oxide produced by inducible NOS during severe infection can contribute to nitrosative stress, epithelial injury, and inflammation [23,24,25].

In contrast, exogenous topical nitric oxide released from NONS acts locally and transiently within the gel at the mucosal surface, reaching virucidal concentrations without activating cellular NOS pathways or causing systemic inflammation [22,26,27,28,29]. This distinction between endogenous overproduction and controlled exogenous delivery is important for therapeutic safety.

The antiviral activity of NONS aligns with well-established redox-dependent mechanisms of nitric oxide. Nitric oxide and its reactive derivatives modify redox-sensitive cysteine residues and disrupt disulfide-stabilized structures that are essential for viral infectivity. In SARS-CoV-2, S-nitrosylation can affect cysteine-dependent viral proteases (3CLpro, PLpro) and destabilize disulfide-rich regions of the spike protein required for ACE2 binding and fusion [23,24,25]. Similar mechanisms likely contribute to influenza virus inactivation, as the glycoproteins hemagglutinin (HA) and neuraminidase (NA) contain multiple conserved cysteine residues whose modification can inhibit fusion and virion release [1,30]. In paramyxoviruses, including RSV, HPIV-3, and hMPV, the cysteine-rich fusion and attachment proteins depend on disulfide stability for receptor interaction and membrane fusion; nitrosylation disrupts these processes, thereby effectively blocking viral entry [31,32,33,34]. Even non-enveloped viruses such as HRV-14, typically resistant to disinfectants, rely on cysteine proteases (2Apro, 3Cpro), whose catalytic residues are susceptible to nitric-oxide-mediated inhibition [22,28,29]. These shared biochemical vulnerabilities provide a unifying mechanistic explanation for NONS’s broad antiviral activity.

Among the viruses tested, influenza viruses exhibited the highest sensitivity, with complete inactivation achieved within 15 s of NONS exposure. This heightened susceptibility may be attributed to the structural and biochemical features of influenza virions. The surface glycoproteins HA and NA are rich in cysteine residues and stabilized by multiple disulfide bonds that are essential for receptor binding, fusion, and virion release. Nitrosative modification or S-nitrosylation of these residues can rapidly disrupt HA’s prefusion conformation and inhibit NA’s enzymatic function, resulting in immediate loss of infectivity [1,2,23]. Furthermore, influenza virions have a smaller size and less glycosylated envelope than SARS-CoV-2 or paramyxoviruses, which may facilitate faster diffusion of nitric oxide and its reactive derivatives across the viral membrane. In contrast, the glycan shielding of the SARS-CoV-2 spike and the heavily glycosylated fusion proteins of RSV and HPIV-3 may offer greater steric protection from nitrosative attack, explaining their comparatively slightly slower inactivation. Collectively, these factors may account for the exceptional rapidity of influenza inactivation by NONS observed in this study.

Because NONS acts through pathogen-independent redox modification rather than specific viral epitopes, its antiviral activity is expected to be resilient to antigenic drift or immune escape. Targeting the upper respiratory tract is a strategic advantage, as the nasal mucosa is the primary site of early viral replication and dissemination and intranasal delivery allows immediate local neutralization. Such fast-acting, localized antivirals may be especially beneficial in settings with limited access to systemic therapeutics or where rapid suppression of transmission is crucial [26,35]. When combined with its favorable safety profile, over-the-counter availability, and intranasal delivery, NONS emerges as an attractive adjunct to existing preventative strategies. Vulnerable populations, including the elderly and immunocompromised, may particularly benefit from its use as an immediate-response antiviral.

While NONS demonstrated good short-term tolerability and showed no evidence of systemic absorption, long-term effects on nasal epithelium biology and the microbiome were not assessed and should be addressed in future clinical studies.

Although the in vitro virucidal assay involves direct mixing of virus suspensions with the formulation under controlled laboratory conditions, the nasal environment contains mucus clearance, airflow dynamics, and host immune factors that may alter contact time and antiviral activity. Therefore, in vitro log-reductions should not be interpreted as direct clinical outcomes. Nevertheless, clinical studies using NONS during the COVID-19 pandemic consistently demonstrated faster viral clearance and reduced transmission, supporting the translation of the observed virucidal activity into real-world benefit.

Together, our in vitro findings align closely with our clinical observations and demonstrate that nitric oxide–mediated viral inactivation translates effectively from bench to bedside. Collectively, these results highlight the translational relevance of NONS and its potential not only for SARS-CoV-2 but also for broader antiviral applications against respiratory pathogens with similar modes of entry, replication, and transmission.

## 5. Conclusions

This study demonstrates the potent and broad-spectrum virucidal activity of a nitric oxide nasal spray (NONS) against a panel of clinically relevant respiratory viruses, including multiple SARS-CoV-2 variants, influenza A (H1N1, H3N2, and H5N1) and B subtypes, respiratory syncytial virus (RSV), human metapneumovirus (hMPV), parainfluenza virus type 3 (HPIV-3), and human rhinovirus 14 (HRV-14). The rapid and consistent inactivation observed across both enveloped and non-enveloped viruses within 15 s to 2 min supports the efficacy of the NONS as a frontline antiviral intervention. Importantly, the NONS was also effective against HRV-14, a non-enveloped virus typically resistant to conventional disinfectants, and against the highly pathogenic avian influenza A (H5N1) virus, which remains a major global health concern due to its pandemic potential. These findings suggest that the NONS acts through a pathogen-independent mechanism involving nitric oxide-mediated modification of viral proteins essential for infectivity.

This study provides clear evidence that the NONS can also rapidly inactivate diverse respiratory viruses representing four major viral families, including pathogens for which no approved vaccines or targeted antivirals exist, such as HRV-14, HPIV-3, and hMPV. The ability of the NONS to neutralize both enveloped and non-enveloped viruses within seconds to minutes highlights its potential as an immediate-response antiviral platform with relevance across seasonal, epidemic, and pandemic pathogens and particularly amid continual viral evolution [9,36].

Given its ease of use, targeted topical delivery, favorable safety profile, and over-the-counter availability, the NONS offers a practical and accessible means to reduce viral transmission, particularly during the early stages of infection or in settings where vaccines and systemic antivirals are limited. Its demonstrated efficacy against both emerging SARS-CoV-2 variants and the zoonotic avian influenza H5N1 underscores its importance in outbreak control and pandemic preparedness as a complement to vaccine development. Further clinical studies will help clarify its optimal dosing, duration, and real-world impact across different respiratory pathogens.

## Figures and Tables

**Table 1:** Virucidal activity of nitric oxide nasal spray (NONS) against SARS-CoV-2 variants and other respiratory viruses.

Virus	Treatment	Time	Control Titer (log_10_ CCID_50_/0.1 mL)	Post-Treatment Titer ^c^	Cytotoxicity ^a^	Neutralization ^b^	LRV ^d^	% Reduction
SARS-CoV-2 (WT)	NONS	30 s	4.5	<0.7	None	None	>3.8	>99.98
Alpha	NONS	2 min	4.3	<0.7	None	None	>3.6	>99.97
Beta	NONS	2 min	4.7	<0.7	None	None	>4.0	>99.99
Gamma	NONS	2 min	4.6	<0.7	None	None	>3.9	>99.98
Delta	NONS	2 min	4.5	<0.7	None	None	>3.8	>99.98
Omicron	NONS	2 min	4.4	<0.7	None	None	>3.7	>99.98
XBB 2.0	NONS	2 min	4.6	<0.7	None	None	>3.9	>99.98
H1N1	NONS	30 s	4.5	<0.7	None	None	>3.8	>99.98
H3N2	NONS	30 s	4.4	<0.7	None	None	>3.7	>99.98
H5N1	NONS	30 s	4.6	<0.7	None	None	>3.9	>99.98
Influenza B	NONS	30 s	4.5	<0.7	None	None	>3.8	>99.98
RSV	NONS	2 min	3.5	<0.7	None	None	>2.8	>99.85
hMPV	NONS	2 min	4.4	<0.7	None	None	>3.7	>99.98
HPIV-3	NONS	2 min	4.5	<0.7	None	None	>3.8	>99.98
HRV-14	NONS	2 min	4.2	<0.7	None	None	>3.5	>99.97
HRV-14	70% ethanol (control)	2 min	4.2	4.0	1:10	None	0.2	~37

^a^ Cytotoxicity indicates the highest dilution at which full (80–100%) cytotoxicity was observed. ^b^ Neutralization controls confirmed that, once neutralized, the formulation exhibited no residual antiviral activity and did not affect virus titration. ^c^ Virus titers represent the mean ± SD of three independent experiments (log_10_ CCID_50_ per 0.1 mL). ^d^ LRV (log-reduction value) represents the reduction in virus titer in the test sample compared with the virus control. Detection limit = 0.7 log_10_ CCID_50_/0.1 mL.

## Data Availability

The datasets produced and analyzed during the current study are available from the corresponding author upon reasonable request. Due to the nature of the work and contractual obligations, raw data cannot be deposited in a public repository but can be shared for verification purposes.
